# Ruptured uterus: Frequency, risk factors and feto-maternal outcome: Current scenario in a low-resource setup

**DOI:** 10.1371/journal.pone.0266062

**Published:** 2022-04-08

**Authors:** Saida Abrar, Tahira Abrar, Ehsan Sayyed, Sidra Ali Naqvi

**Affiliations:** 1 Department of Gynecology and Obstetrics, Lady Reading Hospital, Khyber Medical College, Peshawar, Pakistan; 2 Northwest Medical College, Peshawar, Pakistan; 3 Department of Pain Clinic, Shifa International Hospital, Islamabad, Pakistan; Mayo Clinic Minnesota, UNITED STATES

## Abstract

**Introduction:**

Pakistan is among the countries with the highest maternal death rates. Obstetric hemorrhage accounts for 41% of these deaths. Uterine rupture is a grave obstetric emergency with high maternal and neonatal morbidity and mortality. It is important to identify its frequency and associated risk factors to formulate programs for its prevention and management. This study aimed to assess the frequency, associated risk factors, fetomaternal outcomes, and management of women with the ruptured uterus at our hospital.

**Material and methods:**

It was a retrospective study of 206 women to review data collected from cases of uterine rupture managed at the WCTH Bannu, Pakistan from October 2016 to October 2018. A structured proforma was designed and used to extract data from operating theatre registers and the hospital medical records. In our hospital, there is a strong system of maintaining all information of the patients related to demographics, obstetric information, operative notes, and postoperative course during their hospital stay in the patient’s charts. Detailed information on operative procedures is further maintained in the operation theater register and all these registers are checked in the weekly statistical meetings to ensure proper documentation. Data was entered and analyzed in SPSS package version 21 (IBM Corp.; Armonk, NY, USA). Frequency and percentages were calculated for the categorical variables. For inferential statistics, chi-square or Fischer exact tests were used. A p-value of < 0.05 was considered statistically significant.

**Results:**

The overall incidence of the ruptured uterus was 1.71%. The important etiological factors were grand multiparity 62 (35.2%), obstructed/neglected labour 58 (32.9%), injudicious use of Oxytocin 56 (31.8%) and prostaglandins 26 (14.7%), previous cesarean section 35 (19.8%) and previous pelvic surgery (0.5%). Hysterectomy was done in 80.6% of cases, 34 (19.2%) patients underwent uterine repair and 4.5% had bladder repair. The mortality rate was 21%, mainly due to irreversible shock or disseminated intravascular coagulation. Perinatal mortality was 91.4%. Duration of surgery more than two hours and presentation to the hospital at night time was significantly associated with poor maternal outcome (p = 0.00).

**Conclusion:**

Uterine rupture is a preventable obstetric emergency associated with high fetomaternal morbidity and mortality. The main causes were grand multigravidity, obstructed labour, previous C-sections and injudicious use of oxytocin and prostaglandins. Women with prolonged surgery and admission at night time had a poor maternal outcome.

## Introduction

Uterine rupture is “Partial or complete tearing of the uterine wall antenatally or during labour, leading to extrusion of the product of conception (fetus and /or placenta) into the maternal abdomen” [1, 2]. It still continues to be a deadly obstetric situation associated with not only a high risk of maternal and perinatal morbidity and mortality but also grave postoperative complications. The incidence of uterine rupture corresponds to 1 in 920 cases in developing countries of Asia and Africa [3] in comparison to 74 in 10,000 in the developed world [4]. In Pakistan the reported prevalence varies in different cities, being 1.6% in rural areas and 0.74% in big cities [5, 6]. Women in many countries share a dual liability of financially supporting the whole family, doing household activities and raising and caring for their children. Despite their pivotal role in the community, the health requirements of women are never given a priority resulting in high-level maternal morbidity and mortality, especially in developing countries [7].

The high prevalence of uterine rupture is due to low socio-economic factors [8], lack of awareness, poor obstetric practices [9], cultural practices of home deliveries, the greater number of un-booked obstetric emergencies, and lack of access to comprehensive emergency care facilities [2]. Unlike the developed world, where rupture of the scarred uterus is one of the leading causes of uterine rupture, most ruptures in developing countries result from rupture of the unscarred uterus [1, 10]. The major cause in these countries is obstructed labour, especially in rural areas [2, 11, 12]. Other documented risk factors include grand-multiparity, injudicious use of oxytocin for labour augmentation, uterotonic drugs for induction of labour, instrumental delivery, poorly developed health system, and lack of facilities for timely referral to hospital in remote areas [5, 13].

In Pakistan for every 100,000 live births, there are 186 maternal deaths. The rural areas have a much higher Maternal Mortality Ratio (203 per 100,000 live births) than in urban areas (159 per 100,000 live births). Direct maternal deaths are a major cause of death (96%) with obstetric hemorrhage being the most common cause (41%) [14]. This is mainly due to home delivery (95.3%) or delivery by traditional birth attendants (TBAs) [5]. These patients would try to deliver till the end at home or with TBAs, and reach the hospital only when they develop complications of obstructed labour.

The signs and symptoms of a ruptured uterus vary, but initially, they are non-specific, causing a delay in diagnosis and management. This can be avoided by maintaining a high index of suspicion [15, 16]. It is also associated with catastrophic complications, depending on the time between diagnosis and delivery; varying between developing and developed world due to differences in the health care facilities [8, 10, 13]. The documented fetal complications are neonatal intensive care unit (NICU) admission, fetal hypoxia and neonatal death [10, 13]. Maternal consequences include hemorrhage, bladder injury, hypovolemic shock, vesicovaginal fistula (VVF), permanent loss of fertility, and even maternal death [17]. The reported case fatality rate is as high as 30.4% [18]. It has grave social implications in developing countries like Pakistan, where fertility is considered the very essence of womanhood and loss of fertility can lead to divorce [17].

The type of surgical treatment is dependent on the severity and extent of the rupture, hemodynamic status of the mother, future fertility desire, and surgeons’ experience. The available options include total abdominal hysterectomy (TAH) or subtotal abdominal hysterectomy (STAH), repair of the uterus with or without tubal ligation [11, 19]. Uterine repair is usually reserved for those cases with low transverse rupture, without broad ligament, vaginal and cervical extension, hemodynamically stable condition, controllable hemorrhage, and young patients desiring future fertility. Hysterectomy is suitable for hemodynamically unstable patients, unrepairable rupture, and where fertility is not desired [20].

## Patient and methods

### Study setting and population

This was a retrospective review of data collected for all women managed for uterine rupture from October 2016 to October 2018 at the Department of Obstetrics and Gynecology, Women and Children Teaching Hospital (WCTH) Bannu, Pakistan. Bannu is one of the seven divisions of Khyber Pakhtunkhwa province of Pakistan, located in the central south part. It consists of three districts: Bannu, Lakki-Marwat, and North Waziristan, and has a population of 2,656,801 with a male to female ratio of 1.035:1 [21].

WCTH- Bannu is a secondary level hospital with a 60 beds unit of Gynecology and Obstetrics. Annually, about 5600 deliveries are carried out in the hospital. The hospital has an operation room with three obstetricians, six medical officers, one anesthetist, and 20 midwives working in the department of obstetrics. It has become more burdened since 2008, as there has been displacement of more than 1.6 million populations from Waziristan, with the majority of them moving to Bannu due to reprobate activities by the non-state actors. This has resulted in this hospital serving as a referral site for neighboring zones and regions (Waziristan, Karak, Lakki-Marwat).

All women, managed for uterine rupture at WCTH-Bannu, whether it occurred at the hospital or referred, were included in the study.

### Data collection procedures and instrumentation

A structured proforma was used to collect data from the maternity ward, operating theatre registers as well as the hospital medical records office. Information was gathered on socio-demography (age, parity, gestational age, address), clinical features on admission, booking status, time of admission, obstetric variables (injudicious use of syntocinon, forceful delivery attempts at home, history of previous Caesarian section, or other uterine surgery and the place of attempted vaginal delivery), source of referral, intraoperative findings, management at the hospital and postoperative course and associated maternal and fetal complications. We have a system of maintaining detailed records of patients related to demographics, obstetric information and postoperative outcome in the patients’ medical charts. Furthermore, operative details are maintained in operation registers. All this information is further checked in the weekly statistical meetings to ensure proper documentation.

### Exclusion criteria

1. Women referred to WCTH for postoperative care or management of complications due to uterine rupture at other health facilities.
2. Ruptured uterus caused by second-trimester termination of pregnancy.
3. Cases with missing data on outcome variables and demographics.

Data collection was done by two medical officers supervised by a consultant Obstetrician.

### Definitions of study variables

Multigravidas; Women with two to four pregnancies.

Grand multigravidas; Women with five to nine pregnancies.

Great grand multigravidae; Women with 10 or more pregnancies.

Source of admission; recorded from the admission notes in medical charts.

Sepsis; Woman with a diagnosis and management of sepsis in her medical charts.

Wound infection; Woman with a diagnosis of wound infection in her medical charts.

Wound dehiscence; Woman with a diagnosis of wound dehiscence in her medical charts.

Maternal death due to uterine rupture is defined as the death of the mother from uterine rupture itself, its associated complications, or management.

Poor maternal outcome; Women were labeled as having poor outcomes if they had developed one of these complications: genitourinary fistula, sepsis, wound dehiscence, need for ICU admission, or death.

### Maintaining data quality control

Data quality was assured by adequately designing the data collection tool. We conducted a pretest on 5% of the sample size and after that, all the required correction on the collection tool was made. All the data collectors and the supervisors were trained on information regarding data collection tools and methods of data extraction, objectives of the study, ethical issues considerations. The data collection process was supervised by the assigned supervisor for the accuracy of the process.

### Data processing and analysis

After ensuring completeness of the data, it was entered and analyzed in SPSS package version 21 (IBM Corp.; Armonk, NY, USA). We calculated frequency and percentages for the categorical variables while the mean and standard deviation was determined for continuous variables. Statistical comparison was done using a chi-square or Fischer exact test, where appropriate. A p-value of < 0.05 was considered statistically significant. We used tables to present the findings of the study.

### Ethical consideration

We conducted the study after approval from the Ethical Review Committee (ERC) of the Bannu Medical College, reference number; 72-A/BMC/2016. Since we extracted the data from patients’ medical records with no encounter with the patients, the requirements of written informed consent were waived by the ERC. We maintained the confidentiality of all information by coding the information.

## Results

### Prevalence of uterine rupture

During the study period of two years, 12000 deliveries were conducted in the hospital while the number of cases of uterine rupture managed at the hospital was 206; thus overall prevalence rate was 1.71% (1 in 58). The final study population was 176 after excluding thirty cases (response rate of 85%), 19 due to missing information on study variables, and 11 due to the non-availability of charts in the record room.

### Demographic and obstetric characteristics of patients

There were 81 (46%) women in the 31–40 years age group representing the majority, followed by 65 (36.9%) women in the 21–30 years age group. The mean age was 31years. The majority of the women were housewives (97.7%) and residents of Waziristan (52.2%), with only 50 (28.4%) from Bannu. About half the population resided more than 10 km away from the hospital (Table 1). Most cases were multi and grand multigravidas. Most of them (32.9%) had obstructed labour. Only 35 cases (19.8%) occurred in the scarred uterus. Among the seven primigravidas (3.9%) with a ruptured uterus; three of them had injudicious use of misoprostol for induction of labour by TBA in their maternity clinics. Three cases occurred at home; two had a breech presentation, one had a transverse lie with forceful attempts made to deliver babies with injudicious use of syntocinon at home by TBA (called Dai in Pakistan). The cause of the seventh case was unidentified. Most cases (51.7%) occurred in patients more than 40 weeks gestation. About 95.9% of cases occurred either at home attended by TBA or midwives while seven cases occurred when the women were on admission in the hospital.

**Table 1:** Demographic and obstetric characteristics of mothers with ruptured uterus (n = 176).

Variables	Frequency	Percentage (%)
Maternal Age		
< 20	12	6.8
21–30	65	36.9
31–40	81	46.0
> 40	18	10.2
Occupation		
House-wife	172	97.7
Employed outside the home	4	2.2
Address of the patients		
Bannu	50	28.4
Waziristan	92	52.2
Karak	13	7.3
Marwat	21	11.9
Residence >10 km from Hospital		
Yes	105	59.6
No	71	40.3
Gravidity		
Primi gravida	7	3.9
Multi gravida	55	31.2
Grand multi-gravida	62	35.2
Great grand multi-gravida	52	29.5
Gestation in weeks		
Less than 37	8	4.5
37–40	55	31.2
Above 40	91	51.7
Unknown	22	12.5
Place of delivery		
Hospital	7	3.9
Home (TBA)	43	24.4
Mid wives	126	71.5
Risk Factors		
Obstructed/Neglected Labour (+ Injudicious use of Oxytocin)	58	32.9
Injudicious use of Oxytocin	56	31.8
Injudicious use of prostaglandins	26	14.7
Previous Caesarean Section	35	19.8
Previous Pelvic Surgery	1	0.5
History of ANC visit		
No visit	120	68.1
< 4	41	23.3
≥ 4	15	8.5
Type of referral		
Self-referred	37	21.0
Referred by health-care	139	78.9
professional		
Diagnosis on referral		
Uterine rupture	35	19.8
CPD/OL	83	47.1
Mal-presentation	27	15.3
Prolonged second stage of labour	21	11.9
IUFD	10	5.6
Blood pressure on arrival		
Non recordable	102	57.9
<90/60mmhg	48	27.2
≥90/60mmhg	26	14.7
Diagnosis on admission		
Uterine rupture	149	84.6
Other diagnosis	27	15.3
Time of arrival		
Day	144	81.8
Night	32	18.2

TBA; traditional birth attendants, ANC; antenatal checkup, CPD; cephalopelvic disproportion, OL; obstructed labour, IUFD; intrauterine fetal death

Among the seven cases of uterine rupture at the hospital, five were great grand multipara and two were multipara. Among them, two women were previous two C-sections and had used oral misoprostol advised by midwives for induction of labour. They developed complications of ruptured uterus soon after admission. Three women presented with history of augmentation in the community settings and were complicated by obstructed labour and subsequently uterine rupture at the hospital. Two women presented in advanced labour with transverse lie and were complicated by a uterine rupture in the hospital. Six babies were delivered alive among these seven cases out of which one resulted in early neonatal death. One was a fresh stillbirth. Among the 169 cases presenting with ruptured uterus, 22 women had previous one C-section, nine had previous two C-sections and all of them had a history of augmentation using repeated doses of intramuscular and intravenous oxytocin. Two women had previous three C-sections and one had a history of myomectomy one year ago and developed a ruptured uterus after a trial of labour at home. The remaining patients were mostly induced or augmented with misoprostol and/ or oxytocin injudiciously by midwives or TBAs. Only 14 babies were delivered alive and four of them resulted in early neonatal deaths, while one died on day nine.

Most of the cases (78.9%) were referred by health care professionals. The majority had no antenatal visits and only 15 patients (8.5%) had more than four antenatal visits. Most arrived in the daytime with hypovolemic shock “Table 1”.

### Clinical presentations and management options

The most frequent presenting symptoms were; abdominal pain (58.5%), cessation of labor (52.2%), and vaginal bleeding (39.2%), while the commonest presenting signs of uterine rupture were abdominal tenderness (79.5%) and easily palpable fetal parts (42%). Hysterectomy was done in 80.6% of cases, while the uterine repair was done in only 34 cases “Table 2”.

**Table 2:** Clinical presentations among cases of uterine rupture/management performed for mothers (n = 176).

Variables	Frequency	Percentage (%)
Clinical presentations		
Abdominal pain	103	58.5
Cessation of labour	92	52.2
Vaginal bleeding	69	39.2
Abdominal tenderness	140	79.5
Easily palpable fetal parts	74	42.0
Uterine contraction present	14	7.9
Palpable defect in uterus	34	19.3
Type of management		
Total Hysterectomy	62	35.2
Subtotal Hysterectomy	80	45.4
Repair of uterus	29	16.4
Repair of uterus with BTL	5	2.8

BTL; bilateral tubal ligation.

### Operative findings and postoperative course

The majority of ruptures were complete involving the anterior lower segment (55.6%). Eight cases had accompanying bladder ruptures while 17.6% had broad ligament hematoma with the worst prognosis. More than half of the cases stayed for more than a week in the hospital, with an average hospital stay of 8.9 days, ranging from 4–24 days. All women received a blood transfusion with the majority receiving more than 2 units of blood “Table 3”.

**Table 3:** Operative findings and postoperative course (n = 176).

Characteristics	Frequency	Percentage (%)
Type of rupture		
Complete	155	88.0
Incomplete	21	11.9
Site of rupture		
Anterior lower segment	98	55.6
Posterior	16	9.0
Lateral	41	23.2
Fundal	21	11.9
Bladder injury	8	4.5
Necrotic edges	50	28.4
Vaginal extension	62	35.2
Broad ligament hematoma	31	17.6
Duration of surgery		
<2 h	99	56.2
≥2 h	77	43.7
Hospital stay		
<7 days	79	44.8
≥7days	97	55.1
Blood transfusion		
No	0	0
1 units	14	7.9
2 units	34	19.3
3 units	67	38.0
4 units	49	27.8
≥5 units	12	6.8
Post op hemoglobin		
>12 g/dl	20	11.3
7–12 g/dl	132	75.0
< 7 g/dl	24	13.6

### Postoperative maternal and fetal complications

The most common complication was anemia (79.65) followed by wound infection, sepsis and VVF. There were 89 ICU admissions and 37 maternal deaths (21%); mainly due to irreversible shock or disseminated coagulation (DIC). There is no blood bank at the hospital and there was a considerable delay in arrangement and transfusion of blood. Perinatal mortality was 91.4% and NICU admission was 11.3% due to low Apgar scores “Table 4”.

**Table 4:** Postoperative maternal and fetal complications (n = 176).

Variables	Frequency	Percentages (%)
Maternal complications		
Anemia and need of transfusion	140	79.5
Vault hematoma	14	07.9
DVT	4	02.2
ICU admission	89	50.5
Sepsis	86	48.8
wound infection	109	61.9
Wound dehiscence	13	07.3
Genitourinary fistula	23	13.0
Death	37	21.0
Fetal complications		
Live birth	20	11.3
Apgar<5 at 1 min	20	11.3
Nursery admission	20	11.3
Total perinatal mortality	161	91.4
Stillbirth	156	88.6
Early neonatal deaths	5	02.8

DVT; deep vein thrombosis, ICU; intensive care unit

### Factors associated with poor maternal outcome

Time of arrival at the hospital and duration of surgery was significantly associated with poor maternal outcomes (p = 0.000). Women with ≥ 2 hours duration of surgery had poor outcomes (62.2%) than those with a duration of < 2 hours (37.8%). Similarly, more women with poor outcomes arrived at night time (26.9%). There was no significant association between gravidity, gestation, place of delivery, and antenatal checkups with the poor maternal outcome “Table 5”.

**Table 5:** Factors associated with poor maternal outcomes (n = 176).

Variables	Poor Maternal outcomes		p-value
	Yes	No	
Gravidity n (%)			0.238^a^
Primigravida	6 (5%)	1(1.8%)	
Multigravida	42(35.3%)	13(22.8%)	
Grandmultigravida	39(32.8%)	23(40.4%)	
Great-grandmultigravida	32(61.5%)	20(35.1%)	
Gestational age n (%)			0.125^a^
Less than 37	2(1.7%)	5(8.8%)	
37–40	40(33.6%)	15(26.3%)	
Above 40	40(33.6%)	22(38.6%)	
Unknown	37(31.1%)	15(26.3%)	
Time of arrival to the hospital n (%)			0.000^b^
Day	87(73.1%)	57(94.7%)	
Night	32(26.9%)	0(0%)	
Duration of surgery n (%)			0.000^b^
< 2 h	45(37.8%)	54(96.7%)	
≥ 2 h	74(62.2%)	3(5.3%)	
Place of delivery n (%)			0.378^a^
Hospital	3(2.5%)	4(7%)	
Home (TBA)	30(25.2%)	13(22.8%)	
Midwives	86(72.3%)	40(70.2%)	
History of ANC visit n (%)			0.181^b^
Yes	34(28.6%)	22(38.6%)	
No	85(71.4%)	35(61.4%)	

TBA; traditional birth attendants, ANC; antenatal checkup.

a; Fischer exact test, b; Chi-square test

## Discussion

The current study showed a high incidence of the ruptured uterus in this part of the country i.e 1.71% (1 in 58), which is higher than that reported from a previous study conducted at the same hospital in 2009 [5]. This could be due to the increased workload on the hospital due to military operations in Waziristan, displacing millions of people. This was also much higher than the reported incidence in other cities of Pakistan [5, 6, 22, 23]. However, lower incidences have been reported in other developing countries like Tanzania (0.22%) and India (0.057%) [11, 24]. The reported incidence of uterine rupture was 12 in 36,000 births in developed Countries and these occurred mainly in patients with scarred uteri [10]. This could be attributed to the improved level of obstetric care in these countries.

Most patients in our study (46%) were in the 31–40 years age group followed by 36.9% in the 21–30 years age group. Similar results were found in another local study from Pakistan, where 47% of participants were in the age group 31–35 years and 38.2% in 26–30 years [25]. Similar to findings of other studies, most participants in the current study were housewives (97.7%) [5], unbooked (68.1%) [22] and mostly referred cases (78.9%) [5, 21, 25] from remote areas of North Waziristan (52.2%), especially at night time [5].

In Pakistan, only 50% of women receive ANC and more than 80% of the deliveries are home deliveries, mainly conducted by the TBAs [26]. The TBA’s work is unsupervised with a lack of any backup support [26]. In this study, 95.9% of cases were delivered by TBAs or midwives, either at home or in their maternity homes. The main reason why the women preferred home or Dai delivery was the high rate of C-Section in the hospital. These untrained people would try to deliver each patient vaginally without being able to recognize signs of obstructed labor, resulting in a ruptured uterus. Similar findings were observed in other studies, strengthening the results of our study [5, 13, 26, 27].

Only 19.8% of cases were correctly diagnosed as uterine rupture by referring health professionals as the majority of the cases were initially managed and referred by unskilled midwives and TBAs. It is also possible that uterine rupture occurred on the way to the hospital as 59.6% of women were residing more than 10 kilometers away from the hospital. Residence away from the hospital with a poor emergency transport system is a documented risk factor for a ruptured uterus [5]. Pakistan is one of those unfortunate countries with no proper emergency transport system, particularly in rural areas. The previous study had more patients residing more than 10 kilometers away from the hospital, suggesting some improvement in the transport system.

There were seven cases of uterine rupture at WCTH-Bannu, all of them recovered with no adverse outcome. This could be due to the timely diagnosis and management of these cases. Like findings of other studies, neglected and obstructed labour was the leading cause of ruptured uterus (64.7%) while rupture of scarred uterus accounted for 19.8% of cases [6, 28]. In all cases, oxytocin was used injudiciously. However, in contrast to our study, Malik HS and others found previous cesarean scar as the most common cause (53.39%) [22]. This could be due to the fact that patients with previous C-section scars were delivered by elective repeat C-section at this hospital due non-availability of facilities for intrapartum monitoring (cardiotocography) and shortage of consultants and other medical staff. Injudicious use of prostaglandins was the other most common cause. Among the 26 cases, 19 women (10.7%) had misoprostol used in large than recommended doses for induction of labour, either orally, vaginally or both. This is much higher than the figures reported for cases of uterine rupture with misoprostol use in other studies [29, 30].

Most affected were multigravidas and great grand multigravidas, with complete rupture involving the anterior lower segment as the most common intraoperative finding which is in line with the findings in other studies [6, 22]. The majority of the women had a hysterectomy as an emergency life-saving surgery, with STAH being performed in 45.4% of cases. This high frequency of hysterectomy could be due to the large number of women who presented with unstable hemodynamic conditions and the high number of obstructed labour cases; thus performing this as an emergency lifesaving surgery in the majority of women. Uterine repair was done in only 19.2% of cases. This was in contrast to the findings in the study by Malik HS [21], but was comparable with that in the study by Rizwan et al. [6]. Uterine repair without tubal ligation was performed in only 5 women desiring future fertility. The percentage is much lower than 33.3% recorded in a study by Kankika et al. but comparable with 5.9% in a study by Ahmed et al. [28, 31].

Most of the women were anemic postoperatively requiring blood transfusion, while 50.5% required ICU admission, depicting the worst health condition at the time of referral. Thirteen percent (13%) of the cases were complicated by genitourinary fistulas comprising 21 cases of VVF and two cases of ureterovaginal fistulas. These findings are comparable to those of Ahmed et al. [28] but are in contrast to the findings by Qazi Q et al. [5] and Aliyu et al. [2]. This could be due to the high incidence of obstructed labour cases in our study compared to the other studies.

Maternal mortality in this study was 21% mainly due to DIC and sepsis. This is much higher than reported in other studies [5, 6, 21]. Perinatal mortality was 91.4%, while 11.3% of the fetuses were delivered alive with low Apgar scores and required neonatal intensive care unit (ICU) admissions. These high maternal and perinatal mortality is an indication of the poor healthcare delivery system and maternity services in this part of the country. The total maternal deaths in the hospital during the study period was 80, with ruptured uterus contributing 46.2% (37/80) of these deaths.

This study showed poor maternal outcomes were associated with prolonged surgery (≥ 2 hours) (p = 0.000). About 62.2% of women with ≥ 2 hours operation time resulted in poor maternal outcomes. Similar findings were observed in other studies [28]. This may be due to the hemodynamically unstable condition of the patient and the complex nature of the surgery. Furthermore more women who developed complications presented at night time. This could be a lack of availability of trained staff and difficulties in timely availability of blood at night time. However, gestation, gravidity, place of delivery, and antenatal checkups were not significantly associated with poor maternal outcomes.

This is the only study carried out in this hospital after a massive increase in the number of patients due to internally displaced people, resulting from the military operation against the Taliban in Waziristan. There is a clear increase in the prevalence of obstetric emergencies, evident by the high prevalence of ruptured uterus, with no increased support in medical facilities from the government to tackle the situation.

The limitations of the study are its retrospective design and sample size. In addition, many patients were excluded because of the non-availability of data on the study variables and missing charts. This was a single-center study so its results cannot be generalized to the whole population. Future prospective multicenter studies are required to alleviate these limitations.

## Conclusions

This study reveals an alarmingly high rate of the ruptured uterus and associated maternal mortality in this part of the country. The main factors are grand and great grand-multigravidity, lack of antenatal care, neglected or obstructed labour, injudicious use of oxytocin and misoprostol, poor emergency transport and referral system, and deliveries by TBAs outside the hospital in unsafe locations.

Thus, serious efforts are needed to strengthen the available health facilities. A focused female education program should be promoted and educated women should be empowered to reduce gender inequality and improve their socio-economic status. Efforts should be made to improve communication, referral, and emergency transport systems and universal access to emergency obstetric care. Health education programs to educate women about complications associated with unbooked pregnancies, grand-multiparity, and unsupervised home deliveries should be instituted. Similarly, TBAs should be educated on the importance of early referrals, judicious use of oxytocin, especially in multiparas as well as careful labour management. In addition, their services should be constantly supervised. By these means, the incidence of uterine rupture and its associated sequelae can be significantly reduced.

## Supporting information

S1 File (SAV)
